# Dibutyl­ammonium bis­(hydrogen methylphosphonato-κ*O*)triphenylstannate(IV)

**DOI:** 10.1107/S1600536812038445

**Published:** 2012-09-19

**Authors:** Tidiane Diop, Libasse Diop, Djibril Fall, Arie van Lee

**Affiliations:** a Laboratoire de Chimie Minerale et Analytique, Département de Chimie, Faculté des Sciences et Techniques, Université Cheikh Anta Diop, Dakar, Senegal; b Laboratoire de Chimie Organique et Therapeutique, Département de Pharmacie, Faculté de Medecine, de Pharmacie et d’Odontostomatologie, Université Cheikh Anta Diop, Dakar, Senegal; c Institut Européen des Membranes, Université de Montpellier II, 34000 Montpellier, France

## Abstract

The asymmetric unit of the title organotin salt, (C_8_H_20_N)[Sn(C_6_H_5_)_3_(CH_4_O_3_P)_2_], contains two dibutyl­ammonium cations and two stannate(IV) anions consisting each of two monodentately bonding methyl hydrogenphosphate groups attached to an Sn(C_6_H_5_) unit. The overall coordination environment of the two Sn^IV^ atoms is trigonal–bipyramidal defined by three phenyl C atoms in equatorial positions and two methyl hydrogenphosphate O atoms at the apical sites. In the crystal, the stannate(IV) anions are linked to each other *via* pairs of short O—H⋯O hydrogen bonds, leading to an infinite chain extending parallel to the *b*-axis direction. Neighbouring chains are linked by N—H⋯O hydrogen bonds involving the butyl­ammonium cations, giving a two-dimensional structure parallel to the *ab* plane. The crystal under investigation was found to be twinned by reticular merohedry with twin fractions of 0.5342 (7):0.4658 (7).

## Related literature

For general background to and applications of tin(IV) compounds, see: Davies *et al.* (2008 ▶); Gielen (2002 ▶); Molloy *et al.* (1984 ▶). For related structures, see: Adair *et al.* (2003 ▶); Chunlin *et al.* (2008 ▶); Diop *et al.* (2002 ▶, 2011 ▶); Gueye *et al.* (2011 ▶); Sow *et al.* (2012 ▶). For details of the use of constraints and restraints during the structure refinement, see: Cooper *et al.* (2010 ▶). For background to the weighting schemes used in the refinement, see: Prince (1982 ▶); Watkin (1994 ▶).
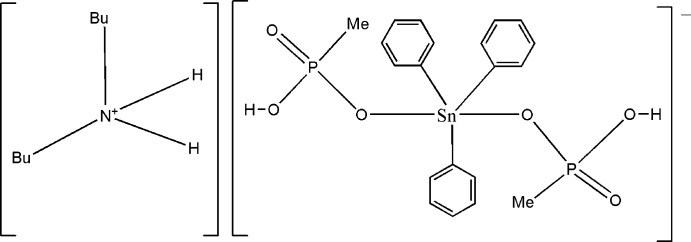


## Experimental

### 

#### Crystal data

(C_8_H_20_N)[Sn(C_6_H_5_)_3_(CH_4_O_3_P)_2_]
*M* *_r_* = 670.28
Monoclinic, 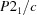

*a* = 16.1963 (8) Å
*b* = 18.9088 (8) Å
*c* = 21.1989 (11) Å
β = 93.220 (4)°
*V* = 6482.0 (5) Å^3^
*Z* = 8
Mo *K*α radiation
μ = 0.93 mm^−1^
*T* = 175 K
0.30 × 0.25 × 0.10 mm

#### Data collection

Oxford Diffraction Gemini diffractometer
Absorption correction: multi-scan (*CrysAlis PRO*; Agilent, 2010 ▶) *T*_min_ = 0.961, *T*_max_ = 1.000
64843 measured reflections
26060 independent reflections
17225 reflections with *I* > 2σ(*I*)
*R*_int_ = 0.085

#### Refinement

*R*[*F* ^2^ > 2σ(*F* ^2^)] = 0.067
*wR*(*F* ^2^) = 0.067
*S* = 0.99
17225 reflections
686 parameters
9 restraints
H-atom parameters constrained
Δρ_max_ = 1.28 e Å^−3^
Δρ_min_ = −1.66 e Å^−3^

### 

Data collection: *CrysAlis PRO* (Agilent, 2010 ▶); cell refinement: *CrysAlis PRO*; data reduction: *CrysAlis PRO*; program(s) used to solve structure: *Superflip* (Palatinus & Chapuis, 2007 ▶); program(s) used to refine structure: *CRYSTALS* (Betteridge *et al.*, 2003 ▶); molecular graphics: *OLEX2* (Dolomanov *et al.*, 2009 ▶); software used to prepare material for publication: *CRYSTALS* and *publCIF* (Westrip, 2010 ▶).

## Supplementary Material

Crystal structure: contains datablock(s) global, I. DOI: 10.1107/S1600536812038445/wm2676sup1.cif

Structure factors: contains datablock(s) I. DOI: 10.1107/S1600536812038445/wm2676Isup2.hkl

Additional supplementary materials:  crystallographic information; 3D view; checkCIF report

## Figures and Tables

**Table 1:** Selected bond lengths (Å)

Sn1—O101	2.175 (4)
Sn1—O106	2.188 (3)
Sn1—C111	2.128 (6)
Sn1—C117	2.137 (6)
Sn1—C123	2.125 (5)
Sn2—O201	2.188 (3)
Sn2—O206	2.169 (4)
Sn2—C211	2.125 (6)
Sn2—C217	2.126 (6)
Sn2—C223	2.134 (6)

**Table 2:** Hydrogen-bond geometry (Å, °)

*D*—H⋯*A*	*D*—H	H⋯*A*	*D*⋯*A*	*D*—H⋯*A*
O103—H1031⋯O109^i^	0.85	1.73	2.582 (10)	180 (1)
O108—H1081⋯O104^ii^	0.85	1.64	2.490 (10)	180 (1)
O203—H2031⋯O209^iii^	0.85	1.63	2.478 (10)	180 (1)
O208—H2081⋯O204^iv^	0.85	1.71	2.561 (10)	180 (1)
N10—H101⋯O209^iii^	0.89	1.90	2.772 (10)	165 (1)
N10—H102⋯O109	0.90	1.92	2.777 (10)	160 (1)
N20—H201⋯O204^iv^	0.89	1.95	2.780 (10)	154 (1)
N20—H202⋯O104	0.90	1.88	2.756 (10)	167 (1)

Symmetry codes: (i) 
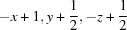
; (ii) 
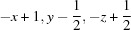
; (iii) 
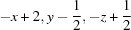
; (iv) 
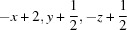
.

**Fig. 1.**
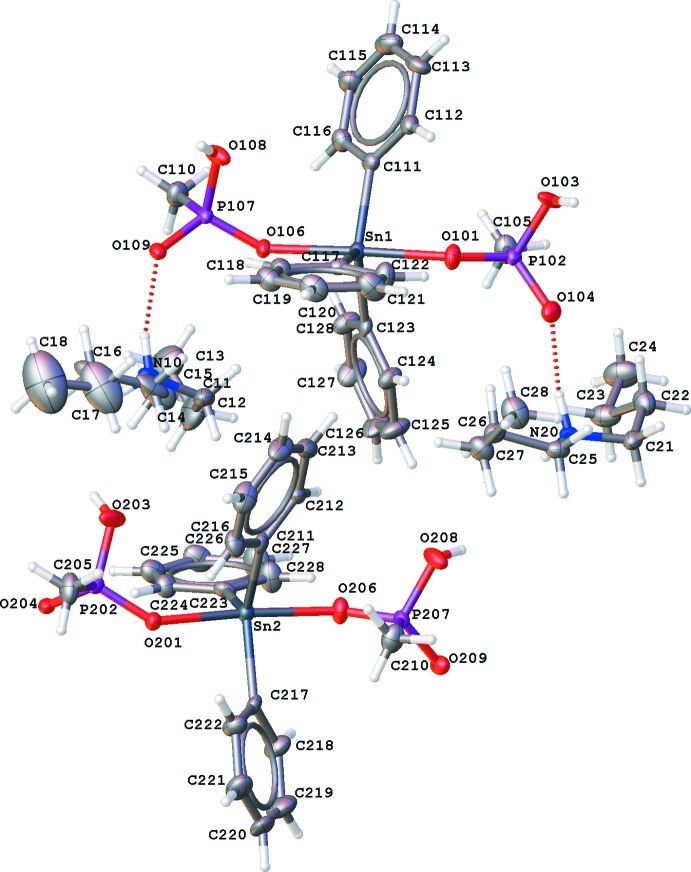
The two pairs of molecular entities in the title compound with anisotropic displacement parameters drawn at the 30% probability level. H atoms are shown as spheres of arbitary radius.

**Fig. 2.**
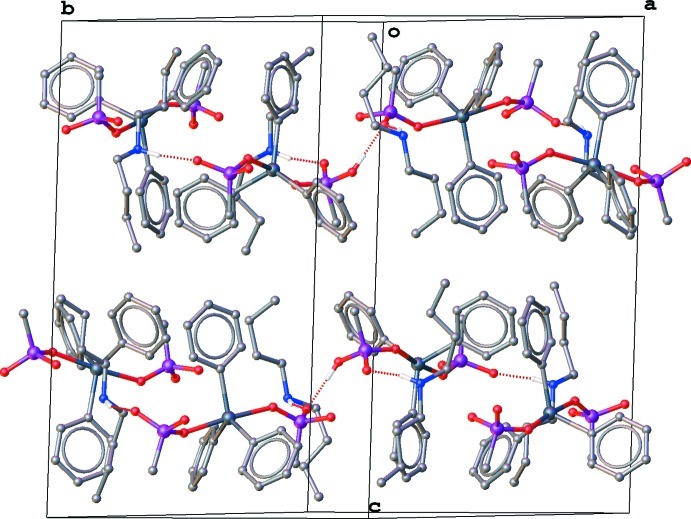
The crystal packing of the title compound. Hydrogen bonds are shown as dashed lines.

## supplementary crystallographic information

### Comment

Organotin(IV) complexes are extensively studied due to their industrial applications as well as for their biocidal properties (Molloy *et al.*, 1984; Gielen, 2002; Davies *et al.*, 2008). Our group has conducted research on SnMe_3_ and SnPh_3_ residues containing derivatives with mono- and polybasic oxyanions such as C_2_O_4_^2-^ (Gueye *et al.*, 2011; Sow *et al.*, 2012) and PhP(H)O^2-^ (Diop *et al.*, 2011). Here we report the structure of the title compound, [(C_4_H_9_)_2_NH_2_]^+^[Sn(C_6_H_5_)_3_(CH_3_PO_2_OH)_2_]^-^, (I).

The asymmetric unit of compound (I) is illustrated in Fig. 1. It consists of two dibutylammonium cations and two organotin complexes consisting of two monodentate MePO_3_H^-^ anions bonded to SnPh_3_; the Sn^IV^ atoms exhibit a *trans* trigonal bipyramidal coordination environment consisting of three phenyl carbon atoms and two MePO_3_H^-^ oxygen atoms. The trigonal plane of the Sn atoms is defined by the three phenyl groups whereas the axial positions are occupied by oxygen atoms from the methyl hydrogenphosphate tetrahedra. A similar arrangement around tin(IV) has been observed in the crystal structure of {[(CH_3_)_3_Sn]_4_(O_3_PPh)_2_}_n_ (Chunlin *et al.*, 2008). The sums of the angles at the tin(IV) positions by the *ipso*-carbons atoms [119.9 (2)°, 121.2 (2)°, 118.8 (2)°, and 122.4 (2)°, 119.0 (2)° and 118.6 (2)° for the two Sn^IV^ entities] are 359.9° and 360.0°, respectively; the corresponding axial O101—Sn1—O106 and O201—Sn2—O206 angles are 174.71 (14)° and 174.15 (14)°, respectively, indicating a nearly perfect *trans* trigonal bipyramidal arrangement. The Sn—C bond lengths are almost identical within the experimental error (Sn1—C111: 2.128 (6) Å; Sn1—C117: 2.137 (6) Å; Sn1—C123 2.125 (5) Å; Sn2—C211: 2.125 (6) Å; Sn2—C217: 2.126 (6) Å; Sn2—C223 2.134 (6) Å] and lie in the range reported for related structures (Gueye *et al.*, 2011). The two axial Sn—O distances, [Sn1—O101 2.175 (4) Å; Sn1—O106 2.188 (3) Å; Sn2—O201 2.188 (3) Å; Sn2—O206 2.169 (4) Å] are in the range of axial Sn—O distances (2.165 (4) and 2.434 (4) Å) observed in *catena*-trimethyltin(IV) methylphosphonate, [MePO_3_HSnMe_3_] (Diop *et al.*, 2002), but are longer than the axial Sn—O distances [2.116 (2) Å and 2.132 (3) Å] observed in *catena*-(µ2-phenylphosphinato O, *O*')-chlorido-tin(II) (Adair *et al.*, 2003). The geometry at the P sites is a distorted tetrahedron with bond angles ranging from 102.7 (3)° for O208—P207—C210 to 114.2 (2) for O201—P202—O204.

The stannate(IV) anions [(MePO_3_H)_2_SnPh_3_]^-^ are linked by pairs of short O—H···O hydrogen bonds, involving the hydroxy group of the methyl hydrogenphosphate unit, and thus forming an infinite chain (Table 1, Fig. 2) along the *b*-direction. In the crystal, neighbouring chains are linked by N—H···O hydrogen bonds *via* the Bu_2_NH_2_^+^ cations, forming a supramolecular structure parallel to the *ab* plane.

### Experimental

An ethanolic solution containing 0.30 g (1.20 mmol) of Bu_2_NH_2_MePO_3_H (obtained from an aqueous mixture of dibutylamine and MeP(O)(OH)_2_ in water in a 1:1 ratio) and triphenyltin(IV) chloride (SnPh_3_Cl) 2.25 g (0.66 mmol) was stirred at room temperature for more than one hour. After 96 h of slow evaporation of the solution, colourless crystals of the title compound (yield: 78%; m.p: 250°) suitable for X-ray structure determination were obtained within the remaining solvent. The powder obtained after complete solvent evaporation has the formula Bu_2_NH_2_Cl according to its infrared spectrum.

### Refinement

H atoms were all located in a difference Fourier map, but those attached to C atoms were repositioned geometrically. They were initially refined with soft restraints on the bond lengths and angles to regularize their geometry (C—H bond lengths in the range 0.93–0.98 Å, N—H bond length 0.89 Å, O—H bond length 0.85 Å) and *U*_iso_(H) (in the range 1.2–1.5 times *U*_eq_ of the parent atom), after which the positions were refined with riding constraints (Cooper *et al.*, 2010).

The large diplacement ellipsoids and deviating C—C distances of the dibutylammonium cations indicated slight displacement disorder, which, however could not be resolved in difference Fourier maps. The dibutylammonium cations were therefore regularized and refined with soft distance and angle restraints.

The crystal under investigation was found to be twinned by reticular merohedry with twin index 7 and twin fractions 0.5342 (7) and 0.4658 (7). The twin symmetry element is a twofold rotation axis along the reciprocal *c*^*^ axis; the pseudo-orthorhombic lattice can be generated by *a*' = *a*, *b*' = *b*, *c*' = 7*c* - *a*.

### Crystal data

**Table:**

(C_8_H_20_N)[Sn(C_6_H_5_)_3_(CH_4_O_3_P)_2_]	*F*(000) = 2768
*M**_r_* = 670.28	*D*_x_ = 1.374 Mg m^−^^3^
Monoclinic, *P*2_1_/*c*	Mo *K*α radiation, λ = 0.71073 Å
Hall symbol: -P 2ybc	Cell parameters from 7455 reflections
*a* = 16.1963 (8) Å	θ = 1.4–29.2°
*b* = 18.9088 (8) Å	µ = 0.93 mm^−^^1^
*c* = 21.1989 (11) Å	*T* = 175 K
β = 93.220 (4)°	Block, colourless
*V* = 6482.0 (5) Å^3^	0.30 × 0.25 × 0.10 mm
*Z* = 8	

### Data collection

**Table:**

Oxford Diffraction Gemini diffractometer	17225 reflections with *I* > 2.0σ(*I*)
Graphite monochromator	*R*_int_ = 0.085
ω scans	θ_max_ = 29.2°, θ_min_ = 1.4°
Absorption correction: multi-scan (*CrysAlis PRO*; Agilent, 2010)	*h* = −20→22
*T*_min_ = 0.961, *T*_max_ = 1.000	*k* = −24→24
64843 measured reflections	*l* = −28→27
26060 independent reflections	

### Refinement

**Table:**

Refinement on *F*	Primary atom site location: charge-flipping
Least-squares matrix: full	Hydrogen site location: difference Fourier map
*R*[*F*^2^ > 2σ(*F*^2^)] = 0.067	H-atom parameters constrained
*wR*(*F*^2^) = 0.067	Method, part 1, Chebychev polynomial, (Watkin, 1994; *P*rince, 1982) [*w*eight] = 1.0/[A_0_*T_0_(x) + A_1_*T_1_(x) ··· + A_n-1_]*T_n-1_(x)] where A_i_ are the Chebychev coefficients listed belo*w* and x = *F* /*F*max Method = Robust Weighting (*P*rince, 1982) W = [*w*eight] * [1-(delta*F*/6*sigma*F*)^2^]^2^ A_i_ are: 15.1 4.23 12.3 3.23
*S* = 0.99	(Δ/σ)_max_ = 0.003
17225 reflections	Δρ_max_ = 1.28 e Å^−^^3^
686 parameters	Δρ_min_ = −1.66 e Å^−^^3^
9 restraints	

### Fractional atomic coordinates and isotropic or equivalent isotropic displacement parameters (Å^2^)

**Table:**

	*x*	*y*	*z*	*U*_iso_*/*U*_eq_	
Sn1	0.55169 (2)	0.19275 (2)	0.195941 (16)	0.0285	
O101	0.5156 (3)	0.3004 (2)	0.21763 (18)	0.0413	
P102	0.49515 (8)	0.37108 (7)	0.18772 (7)	0.0296	
O103	0.4023 (2)	0.3900 (2)	0.1956 (2)	0.0506	
H1031	0.3906	0.4158	0.2267	0.0600*	
O104	0.5479 (2)	0.4314 (2)	0.2139 (2)	0.0440	
C105	0.5019 (5)	0.3644 (4)	0.1041 (3)	0.0534	
H1051	0.4684	0.3265	0.0876	0.0664*	
H1052	0.4828	0.4075	0.0840	0.0664*	
H1053	0.5576	0.3566	0.0936	0.0664*	
O106	0.5926 (2)	0.08826 (17)	0.16616 (18)	0.0316	
P107	0.57266 (8)	0.01052 (7)	0.16887 (7)	0.0296	
O108	0.4833 (2)	0.0013 (2)	0.1908 (2)	0.0506	
H1081	0.4726	−0.0226	0.2233	0.0601*	
O109	0.6335 (2)	−0.03205 (18)	0.20970 (19)	0.0335	
C110	0.5695 (4)	−0.0236 (4)	0.0899 (3)	0.0528	
H1101	0.5316	0.0027	0.0631	0.1230*	
H1102	0.5532	−0.0721	0.0893	0.1230*	
H1103	0.6233	−0.0204	0.0731	0.1230*	
C111	0.4357 (3)	0.1784 (3)	0.1454 (3)	0.0360	
C112	0.3649 (4)	0.2063 (3)	0.1685 (3)	0.0410	
H1121	0.3669	0.2318	0.2059	0.0493*	
C113	0.2880 (3)	0.1965 (4)	0.1360 (4)	0.0557	
H1131	0.2405	0.2156	0.1530	0.0669*	
C114	0.2816 (5)	0.1595 (4)	0.0803 (4)	0.0654	
H1141	0.2309	0.1530	0.0589	0.0784*	
C115	0.3523 (4)	0.1322 (4)	0.0560 (4)	0.0552	
H1151	0.3485	0.1083	0.0178	0.0671*	
C116	0.4285 (4)	0.1411 (3)	0.0886 (3)	0.0433	
H1161	0.4763	0.1224	0.0724	0.0510*	
C117	0.5635 (3)	0.1714 (3)	0.2950 (3)	0.0332	
C118	0.5865 (3)	0.1055 (3)	0.3186 (3)	0.0399	
H1181	0.5968	0.0690	0.2910	0.0475*	
C119	0.5950 (4)	0.0922 (4)	0.3827 (3)	0.0500	
H1191	0.6097	0.0472	0.3970	0.0600*	
C120	0.5810 (5)	0.1433 (4)	0.4247 (3)	0.0580	
H1201	0.5858	0.1340	0.4680	0.0721*	
C121	0.5580 (5)	0.2094 (5)	0.4041 (3)	0.0633	
H1211	0.5471	0.2444	0.4337	0.0761*	
C122	0.5503 (5)	0.2239 (3)	0.3389 (3)	0.0527	
H1221	0.5360	0.2685	0.3252	0.0635*	
C123	0.6561 (3)	0.2318 (3)	0.1505 (3)	0.0328	
C124	0.7102 (4)	0.2768 (3)	0.1807 (3)	0.0492	
H1241	0.7007	0.2909	0.2218	0.0591*	
C125	0.7801 (4)	0.3024 (4)	0.1519 (4)	0.0679	
H1251	0.8163	0.3331	0.1729	0.0821*	
C126	0.7936 (5)	0.2831 (4)	0.0917 (4)	0.0686	
H1261	0.8414	0.2987	0.0728	0.0820*	
C127	0.7387 (5)	0.2393 (4)	0.0596 (4)	0.0666	
H1271	0.7466	0.2268	0.0181	0.0781*	
C128	0.6701 (4)	0.2132 (4)	0.0889 (3)	0.0501	
H1281	0.6333	0.1824	0.0671	0.0591*	
Sn2	1.06366 (2)	0.19358 (2)	0.300170 (16)	0.0299	
O201	1.1082 (2)	0.09045 (18)	0.33422 (18)	0.0329	
P202	1.08457 (8)	0.01320 (7)	0.33239 (7)	0.0311	
O203	0.9928 (2)	0.0086 (3)	0.3079 (3)	0.0602	
H2031	0.9764	−0.0176	0.2771	0.0723*	
O204	1.1394 (2)	−0.03227 (18)	0.29425 (19)	0.0328	
C205	1.0875 (5)	−0.0203 (4)	0.4117 (4)	0.0628	
H2051	1.0734	−0.0692	0.4117	0.0705*	
H2052	1.0505	0.0052	0.4364	0.0705*	
H2053	1.1426	−0.0154	0.4310	0.0705*	
O206	1.0252 (3)	0.3003 (2)	0.27527 (19)	0.0446	
P207	1.00599 (8)	0.37021 (7)	0.30592 (7)	0.0324	
O208	0.9118 (2)	0.3870 (3)	0.2967 (3)	0.0565	
H2081	0.8950	0.4139	0.2665	0.0666*	
O209	1.0549 (2)	0.4318 (2)	0.2817 (2)	0.0404	
C210	1.0210 (5)	0.3624 (4)	0.3897 (3)	0.0559	
H2101	0.9901	0.3235	0.4048	0.0675*	
H2102	1.0781	0.3551	0.4017	0.0675*	
H2103	1.0031	0.4046	0.4098	0.0675*	
C211	0.9552 (4)	0.1822 (3)	0.3512 (3)	0.0373	
C212	0.8793 (3)	0.2069 (3)	0.3260 (3)	0.0379	
H2121	0.8748	0.2289	0.2863	0.0451*	
C213	0.8088 (4)	0.1984 (4)	0.3591 (4)	0.0554	
H2131	0.7593	0.2165	0.3424	0.0659*	
C214	0.8108 (5)	0.1656 (4)	0.4158 (4)	0.0629	
H2141	0.7629	0.1588	0.4365	0.0745*	
C215	0.8862 (5)	0.1414 (4)	0.4430 (4)	0.0648	
H2151	0.8881	0.1205	0.4829	0.0768*	
C216	0.9571 (5)	0.1492 (3)	0.4095 (3)	0.0501	
H2161	1.0075	0.1329	0.4269	0.0593*	
C217	1.1762 (3)	0.2337 (3)	0.3424 (3)	0.0338	
C218	1.2274 (4)	0.2774 (4)	0.3092 (4)	0.0561	
H2181	1.2114	0.2898	0.2682	0.0670*	
C219	1.3013 (5)	0.3022 (5)	0.3362 (5)	0.0772	
H2191	1.3342	0.3318	0.3134	0.0910*	
C220	1.3251 (5)	0.2840 (4)	0.3964 (4)	0.0646	
H2201	1.3753	0.3008	0.4135	0.0769*	
C221	1.2774 (5)	0.2419 (4)	0.4310 (4)	0.0656	
H2211	1.2948	0.2297	0.4724	0.0781*	
C222	1.2018 (4)	0.2164 (4)	0.4036 (3)	0.0508	
H2221	1.1688	0.1878	0.4277	0.0594*	
C223	1.0585 (3)	0.1674 (3)	0.2021 (3)	0.0353	
C224	1.0816 (4)	0.1013 (3)	0.1805 (3)	0.0437	
H2241	1.0993	0.0676	0.2094	0.0522*	
C225	1.0786 (4)	0.0854 (4)	0.1166 (3)	0.0519	
H2251	1.0967	0.0416	0.1033	0.0613*	
C226	1.0495 (4)	0.1335 (4)	0.0728 (3)	0.0592	
H2261	1.0446	0.1227	0.0298	0.0711*	
C227	1.0266 (6)	0.1998 (5)	0.0932 (3)	0.0733	
H2271	1.0096	0.2344	0.0636	0.0861*	
C228	1.0293 (5)	0.2172 (4)	0.1571 (3)	0.0586	
H2281	1.0113	0.2609	0.1702	0.0690*	
N10	0.7984 (3)	0.0021 (3)	0.2374 (2)	0.0397	
H101	0.8398	−0.0259	0.2269	0.0581*	
H102	0.7505	−0.0165	0.2220	0.0580*	
C11	0.8098 (4)	0.0735 (4)	0.2092 (4)	0.0590	
H111	0.8509	0.0989	0.2359	0.0722*	
H112	0.7582	0.0986	0.2090	0.0721*	
C12	0.8419 (7)	0.0707 (5)	0.1451 (4)	0.1010	
H121	0.8985	0.0577	0.1505	0.1239*	
H122	0.8378	0.1177	0.1294	0.1239*	
C13	0.8007 (6)	0.0261 (6)	0.0961 (4)	0.1139	
H131	0.8020	−0.0213	0.1110	0.1425*	
H132	0.7449	0.0402	0.0875	0.1425*	
C14	0.8478 (6)	0.0293 (6)	0.0368 (4)	0.1091	
H141	0.8216	0.0007	0.0047	0.1306*	
H142	0.9025	0.0127	0.0461	0.1306*	
H143	0.8496	0.0769	0.0227	0.1306*	
C15	0.7993 (4)	0.0052 (5)	0.3066 (3)	0.0659	
H151	0.7482	0.0280	0.3176	0.0786*	
H152	0.8468	0.0330	0.3227	0.0791*	
C16	0.8015 (4)	−0.0687 (5)	0.3348 (3)	0.0731	
H161	0.7513	−0.0929	0.3200	0.0892*	
H162	0.8479	−0.0928	0.3187	0.0889*	
C17	0.8065 (9)	−0.0572 (6)	0.4059 (4)	0.1531	
H171	0.8527	−0.0278	0.4170	0.1878*	
H172	0.7574	−0.0350	0.4182	0.1878*	
C18	0.8169 (12)	−0.1248 (8)	0.4378 (5)	0.2082	
H181	0.8202	−0.1166	0.4821	0.2303*	
H182	0.8661	−0.1472	0.4259	0.2303*	
H183	0.7708	−0.1544	0.4271	0.2303*	
N20	0.6997 (2)	0.4947 (2)	0.2381 (2)	0.0309	
H201	0.7428	0.4776	0.2187	0.0458*	
H202	0.6542	0.4700	0.2261	0.0463*	
C21	0.6841 (4)	0.5698 (3)	0.2174 (3)	0.0445	
H211	0.7364	0.5941	0.2176	0.0532*	
H212	0.6498	0.5929	0.2471	0.0532*	
C22	0.6400 (4)	0.5728 (4)	0.1526 (3)	0.0603	
H221	0.6343	0.6227	0.1417	0.0720*	
H222	0.5852	0.5519	0.1544	0.0721*	
C23	0.6829 (3)	0.5372 (4)	0.1006 (3)	0.0628	
H231	0.6902	0.4872	0.1105	0.0753*	
H232	0.7364	0.5591	0.0965	0.0751*	
C24	0.6310 (5)	0.5448 (5)	0.0389 (3)	0.0877	
H241	0.6585	0.5199	0.0063	0.1319*	
H242	0.6246	0.5938	0.0271	0.1320*	
H243	0.5770	0.5248	0.0428	0.1321*	
C25	0.7138 (3)	0.4836 (3)	0.3079 (3)	0.0403	
H251	0.7670	0.5050	0.3207	0.0479*	
H252	0.6708	0.5084	0.3290	0.0483*	
C26	0.7143 (4)	0.4058 (3)	0.3241 (3)	0.0421	
H261	0.6609	0.3854	0.3110	0.0499*	
H262	0.7570	0.3824	0.3010	0.0505*	
C27	0.7300 (4)	0.3921 (4)	0.3944 (3)	0.0555	
H271	0.7379	0.3418	0.4009	0.0672*	
H272	0.7801	0.4179	0.4089	0.0672*	
C28	0.6607 (5)	0.4144 (5)	0.4341 (4)	0.0701	
H281	0.6113	0.3892	0.4209	0.1071*	
H282	0.6730	0.4054	0.4786	0.1069*	
H283	0.6516	0.4642	0.4276	0.1068*	

### Atomic displacement parameters (Å^2^)

**Table:**

	*U*^11^	*U*^22^	*U*^33^	*U*^12^	*U*^13^	*U*^23^
Sn1	0.03063 (19)	0.02368 (17)	0.0318 (2)	0.00151 (15)	0.00695 (13)	0.00110 (15)
O101	0.057 (2)	0.0268 (18)	0.041 (2)	0.0067 (18)	0.0122 (17)	−0.0018 (17)
P102	0.0247 (7)	0.0243 (6)	0.0398 (7)	0.0022 (5)	0.0031 (5)	−0.0042 (5)
O103	0.0185 (19)	0.065 (3)	0.068 (3)	0.0100 (17)	−0.0051 (18)	−0.036 (2)
O104	0.040 (2)	0.036 (2)	0.057 (3)	0.0020 (17)	0.0118 (19)	−0.0128 (19)
C105	0.072 (4)	0.052 (4)	0.035 (3)	0.010 (3)	0.002 (3)	0.000 (3)
O106	0.0303 (17)	0.0197 (16)	0.045 (2)	0.0014 (14)	0.0067 (15)	0.0019 (15)
P107	0.0235 (6)	0.0232 (6)	0.0423 (8)	0.0003 (5)	0.0040 (5)	0.0047 (5)
O108	0.028 (2)	0.061 (3)	0.063 (3)	0.0038 (19)	0.0038 (19)	0.034 (2)
O109	0.0215 (17)	0.0274 (18)	0.052 (2)	0.0011 (14)	0.0056 (16)	0.0038 (16)
C110	0.062 (4)	0.044 (4)	0.051 (4)	0.005 (3)	−0.002 (3)	−0.011 (3)
C111	0.034 (3)	0.028 (3)	0.046 (3)	−0.001 (2)	−0.001 (2)	0.009 (2)
C112	0.036 (3)	0.029 (3)	0.059 (4)	−0.001 (2)	0.012 (3)	0.004 (2)
C113	0.025 (3)	0.064 (4)	0.078 (5)	0.003 (3)	0.000 (3)	0.017 (4)
C114	0.054 (4)	0.064 (5)	0.075 (6)	−0.014 (4)	−0.021 (4)	0.019 (4)
C115	0.050 (4)	0.056 (4)	0.058 (4)	0.000 (3)	−0.011 (3)	−0.003 (3)
C116	0.046 (3)	0.039 (3)	0.044 (3)	0.006 (3)	0.001 (3)	0.006 (3)
C117	0.027 (3)	0.038 (3)	0.035 (3)	−0.005 (2)	0.003 (2)	−0.001 (2)
C118	0.037 (3)	0.040 (3)	0.043 (3)	0.004 (2)	0.003 (2)	0.002 (3)
C119	0.046 (4)	0.061 (4)	0.044 (4)	0.007 (3)	0.006 (3)	0.013 (3)
C120	0.062 (4)	0.080 (5)	0.033 (3)	0.007 (4)	0.003 (3)	0.010 (3)
C121	0.067 (4)	0.083 (6)	0.040 (4)	0.014 (4)	0.002 (3)	−0.009 (3)
C122	0.076 (5)	0.040 (3)	0.043 (4)	0.007 (3)	0.005 (3)	0.003 (3)
C123	0.039 (3)	0.019 (2)	0.040 (3)	0.004 (2)	0.006 (2)	0.006 (2)
C124	0.053 (4)	0.042 (3)	0.053 (4)	−0.014 (3)	0.011 (3)	−0.013 (3)
C125	0.057 (4)	0.066 (5)	0.082 (5)	−0.032 (4)	0.014 (4)	−0.015 (4)
C126	0.065 (4)	0.049 (4)	0.096 (6)	−0.027 (3)	0.042 (4)	0.002 (4)
C127	0.081 (5)	0.065 (5)	0.058 (5)	−0.018 (4)	0.039 (4)	−0.008 (4)
C128	0.057 (4)	0.054 (4)	0.040 (3)	−0.020 (3)	0.009 (3)	−0.008 (3)
Sn2	0.03160 (19)	0.02667 (18)	0.0314 (2)	0.00085 (15)	0.00178 (13)	−0.00105 (16)
O201	0.0310 (18)	0.0242 (17)	0.043 (2)	0.0006 (14)	−0.0008 (15)	−0.0001 (15)
P202	0.0265 (6)	0.0241 (6)	0.0433 (8)	−0.0018 (5)	0.0064 (6)	−0.0043 (5)
O203	0.028 (2)	0.066 (3)	0.087 (4)	−0.001 (2)	0.007 (2)	−0.051 (3)
O204	0.0223 (17)	0.0257 (18)	0.050 (2)	−0.0015 (14)	0.0027 (16)	−0.0027 (16)
C205	0.091 (6)	0.038 (3)	0.061 (5)	0.011 (4)	0.023 (4)	0.011 (3)
O206	0.063 (3)	0.0279 (19)	0.043 (2)	0.0094 (19)	0.0026 (19)	0.0039 (17)
P207	0.0268 (7)	0.0261 (6)	0.0444 (8)	0.0026 (5)	0.0034 (6)	0.0061 (6)
O208	0.029 (2)	0.059 (3)	0.083 (4)	0.0078 (19)	0.009 (2)	0.034 (3)
O209	0.034 (2)	0.033 (2)	0.053 (3)	−0.0015 (16)	−0.0044 (17)	0.0153 (18)
C210	0.080 (5)	0.046 (4)	0.041 (3)	0.000 (3)	0.005 (3)	−0.005 (3)
C211	0.045 (3)	0.019 (3)	0.049 (3)	0.001 (2)	0.012 (3)	−0.006 (2)
C212	0.031 (3)	0.026 (3)	0.057 (4)	0.003 (2)	0.005 (2)	−0.006 (2)
C213	0.033 (3)	0.043 (4)	0.090 (6)	0.001 (3)	0.005 (3)	−0.016 (4)
C214	0.053 (4)	0.054 (4)	0.087 (6)	−0.010 (3)	0.044 (4)	−0.021 (4)
C215	0.089 (6)	0.055 (4)	0.055 (4)	0.002 (4)	0.037 (4)	−0.004 (3)
C216	0.058 (4)	0.044 (3)	0.050 (4)	0.014 (3)	0.016 (3)	−0.002 (3)
C217	0.041 (3)	0.018 (2)	0.043 (3)	−0.004 (2)	0.008 (2)	−0.003 (2)
C218	0.060 (4)	0.050 (4)	0.058 (4)	−0.021 (3)	0.005 (3)	0.005 (3)
C219	0.064 (5)	0.067 (5)	0.102 (7)	−0.031 (4)	0.012 (4)	−0.002 (5)
C220	0.054 (4)	0.042 (4)	0.096 (6)	−0.018 (3)	−0.014 (4)	−0.016 (4)
C221	0.074 (5)	0.056 (4)	0.063 (5)	−0.013 (4)	−0.019 (4)	−0.009 (4)
C222	0.048 (3)	0.049 (4)	0.054 (4)	−0.013 (3)	−0.007 (3)	0.001 (3)
C223	0.024 (3)	0.049 (3)	0.032 (3)	0.001 (2)	−0.005 (2)	0.003 (2)
C224	0.047 (3)	0.045 (3)	0.039 (3)	0.006 (3)	0.008 (3)	−0.002 (3)
C225	0.047 (4)	0.064 (4)	0.045 (4)	0.007 (3)	0.006 (3)	−0.013 (3)
C226	0.061 (4)	0.084 (5)	0.032 (3)	0.009 (4)	0.000 (3)	−0.010 (3)
C227	0.105 (6)	0.075 (5)	0.039 (4)	0.021 (5)	−0.002 (4)	0.012 (4)
C228	0.083 (5)	0.055 (4)	0.037 (3)	0.018 (4)	0.000 (3)	0.000 (3)
N10	0.022 (2)	0.050 (3)	0.047 (3)	0.0011 (19)	0.0025 (19)	−0.010 (2)
C11	0.037 (3)	0.041 (4)	0.098 (6)	−0.002 (3)	−0.005 (3)	−0.009 (4)
C12	0.146 (10)	0.053 (5)	0.105 (9)	−0.014 (6)	0.022 (7)	0.025 (5)
C13	0.158 (11)	0.091 (8)	0.092 (8)	−0.032 (8)	0.001 (8)	0.030 (6)
C14	0.110 (8)	0.144 (10)	0.075 (7)	0.041 (7)	0.015 (6)	0.034 (7)
C15	0.032 (3)	0.121 (7)	0.045 (4)	0.028 (4)	0.004 (3)	−0.021 (4)
C16	0.033 (3)	0.135 (8)	0.052 (4)	0.011 (4)	0.002 (3)	0.021 (5)
C17	0.186 (14)	0.205 (16)	0.073 (8)	0.077 (12)	0.053 (8)	0.040 (9)
C18	0.29 (3)	0.23 (2)	0.112 (13)	0.098 (19)	0.069 (14)	0.054 (13)
N20	0.0214 (19)	0.032 (2)	0.039 (3)	−0.0033 (16)	0.0043 (17)	−0.0015 (18)
C21	0.036 (3)	0.034 (3)	0.065 (4)	−0.004 (2)	0.010 (3)	−0.001 (3)
C22	0.055 (4)	0.052 (4)	0.075 (5)	0.008 (3)	0.011 (4)	0.017 (4)
C23	0.050 (4)	0.082 (5)	0.056 (5)	−0.013 (4)	−0.003 (3)	0.018 (4)
C24	0.081 (6)	0.115 (8)	0.064 (6)	−0.023 (6)	−0.017 (4)	0.018 (5)
C25	0.031 (3)	0.052 (3)	0.038 (3)	−0.005 (2)	0.001 (2)	−0.005 (3)
C26	0.039 (3)	0.042 (3)	0.045 (3)	−0.003 (2)	0.007 (2)	−0.004 (3)
C27	0.053 (4)	0.066 (4)	0.047 (4)	0.010 (3)	−0.004 (3)	0.004 (3)
C28	0.082 (5)	0.081 (5)	0.048 (4)	0.018 (4)	0.015 (4)	0.008 (4)

### Geometric parameters (Å, º)

**Table:**

Sn1—O101	2.175 (4)	C214—C215	1.398 (12)
Sn1—O106	2.188 (3)	C215—H2151	0.933
Sn1—C111	2.128 (6)	C215—C216	1.391 (10)
Sn1—C117	2.137 (6)	C216—H2161	0.930
Sn1—C123	2.125 (5)	C217—C218	1.390 (8)
Sn2—O201	2.188 (3)	C217—C222	1.378 (9)
Sn2—O206	2.169 (4)	C218—H2181	0.923
Sn2—C211	2.125 (6)	C218—C219	1.380 (10)
Sn2—C217	2.126 (6)	C219—H2191	0.926
Sn2—C223	2.134 (6)	C219—C220	1.357 (12)
O101—P102	1.507 (4)	C220—H2201	0.927
P102—O103	1.563 (4)	C220—C221	1.353 (11)
P102—O104	1.511 (4)	C221—H2211	0.935
P102—C105	1.787 (6)	C221—C222	1.410 (9)
O103—H1031	0.850	C222—H2221	0.933
C105—H1051	0.953	C223—C224	1.388 (8)
C105—H1052	0.962	C223—C228	1.405 (9)
C105—H1053	0.953	C224—H2241	0.920
O106—P107	1.507 (3)	C224—C225	1.384 (9)
P107—O108	1.554 (4)	C225—H2251	0.929
P107—O109	1.508 (4)	C225—C226	1.365 (10)
P107—C110	1.793 (7)	C226—H2261	0.933
O108—H1081	0.850	C226—C227	1.384 (11)
C110—H1101	0.951	C227—H2271	0.937
C110—H1102	0.954	C227—C228	1.393 (10)
C110—H1103	0.963	C228—H2281	0.924
C111—C112	1.376 (8)	N10—H101	0.893
C111—C116	1.395 (9)	N10—H102	0.896
C112—H1121	0.927	N10—C11	1.493 (9)
C112—C113	1.401 (9)	N10—C15	1.466 (8)
C113—H1131	0.938	C11—H111	0.975
C113—C114	1.373 (11)	C11—H112	0.960
C114—H1141	0.924	C11—C12	1.483 (12)
C114—C115	1.381 (11)	C12—H121	0.950
C115—H1151	0.926	C12—H122	0.950
C115—C116	1.390 (9)	C12—C13	1.469 (8)
C116—H1161	0.933	C13—H131	0.950
C117—C118	1.386 (8)	C13—H132	0.950
C117—C122	1.386 (8)	C13—C14	1.508 (8)
C118—H1181	0.926	C14—H141	0.950
C118—C119	1.380 (9)	C14—H142	0.950
C119—H1191	0.930	C14—H143	0.950
C119—C120	1.342 (10)	C15—H151	0.973
C120—H1201	0.933	C15—H152	0.977
C120—C121	1.368 (11)	C15—C16	1.520 (12)
C121—H1211	0.936	C16—H161	0.969
C121—C122	1.408 (9)	C16—H162	0.958
C122—H1221	0.917	C16—C17	1.520 (8)
C123—C124	1.357 (8)	C17—H171	0.950
C123—C128	1.383 (8)	C17—H172	0.950
C124—H1241	0.930	C17—C18	1.453 (9)
C124—C125	1.403 (9)	C18—H181	0.950
C125—H1251	0.922	C18—H182	0.950
C125—C126	1.356 (11)	C18—H183	0.950
C126—H1261	0.938	N20—H201	0.892
C126—C127	1.368 (11)	N20—H202	0.897
C127—H1271	0.928	N20—C21	1.504 (7)
C127—C128	1.392 (9)	N20—C25	1.500 (7)
C128—H1281	0.937	C21—H211	0.964
O201—P202	1.510 (4)	C21—H212	0.968
P202—O203	1.549 (4)	C21—C22	1.513 (9)
P202—O204	1.504 (4)	C22—H221	0.974
P202—C205	1.795 (7)	C22—H222	0.976
O203—H2031	0.850	C22—C23	1.495 (7)
C205—H2051	0.951	C23—H231	0.975
C205—H2052	0.949	C23—H232	0.969
C205—H2053	0.965	C23—C24	1.521 (7)
O206—P207	1.513 (4)	C24—H241	0.965
P207—O208	1.560 (4)	C24—H242	0.965
P207—O209	1.515 (4)	C24—H243	0.960
P207—C210	1.785 (7)	C25—H251	0.976
O208—H2081	0.850	C25—H252	0.968
C210—H2101	0.956	C25—C26	1.510 (9)
C210—H2102	0.955	C26—H261	0.973
C210—H2103	0.959	C26—H262	0.976
C211—C212	1.393 (8)	C26—C27	1.520 (9)
C211—C216	1.384 (9)	C27—H271	0.969
C212—H2121	0.939	C27—H272	0.981
C212—C213	1.383 (9)	C27—C28	1.501 (10)
C213—H2131	0.923	C28—H281	0.960
C213—C214	1.352 (11)	C28—H282	0.967
C214—H2141	0.920	C28—H283	0.961
			
O101—Sn1—O106	174.71 (14)	C215—C216—H2161	119.7
O101—Sn1—C111	89.15 (19)	C211—C216—H2161	118.5
O106—Sn1—C111	90.75 (18)	Sn2—C217—C218	121.3 (5)
O101—Sn1—C117	88.80 (18)	Sn2—C217—C222	121.2 (4)
O106—Sn1—C117	95.83 (18)	C218—C217—C222	117.5 (6)
C111—Sn1—C117	119.9 (2)	C217—C218—H2181	119.0
O101—Sn1—C123	90.03 (17)	C217—C218—C219	121.3 (7)
O106—Sn1—C123	85.50 (16)	H2181—C218—C219	119.7
C111—Sn1—C123	121.2 (2)	C218—C219—H2191	120.0
C117—Sn1—C123	118.8 (2)	C218—C219—C220	119.8 (7)
Sn1—O101—P102	142.8 (2)	H2191—C219—C220	120.2
O101—P102—O103	110.3 (3)	C219—C220—H2201	118.5
O101—P102—O104	114.0 (2)	C219—C220—C221	121.3 (7)
O103—P102—O104	108.4 (2)	H2201—C220—C221	120.2
O101—P102—C105	109.3 (3)	C220—C221—H2211	120.2
O103—P102—C105	103.6 (3)	C220—C221—C222	119.1 (8)
O104—P102—C105	110.7 (3)	H2211—C221—C222	120.7
P102—O103—H1031	118.3	C221—C222—C217	121.0 (6)
P102—C105—H1051	110.6	C221—C222—H2221	119.0
P102—C105—H1052	110.0	C217—C222—H2221	120.1
H1051—C105—H1052	108.3	Sn2—C223—C224	122.3 (4)
P102—C105—H1053	110.7	Sn2—C223—C228	119.9 (5)
H1051—C105—H1053	109.0	C224—C223—C228	117.8 (6)
H1052—C105—H1053	108.3	C223—C224—H2241	118.9
Sn1—O106—P107	143.2 (2)	C223—C224—C225	121.6 (6)
O106—P107—O108	109.0 (2)	H2241—C224—C225	119.5
O106—P107—O109	114.1 (2)	C224—C225—H2251	119.8
O108—P107—O109	111.0 (2)	C224—C225—C226	120.8 (6)
O106—P107—C110	108.1 (3)	H2251—C225—C226	119.4
O108—P107—C110	105.1 (3)	C225—C226—H2261	121.8
O109—P107—C110	109.1 (3)	C225—C226—C227	118.7 (6)
P107—O108—H1081	122.5	H2261—C226—C227	119.5
P107—C110—H1101	110.7	C226—C227—H2271	119.9
P107—C110—H1102	110.6	C226—C227—C228	121.6 (7)
H1101—C110—H1102	109.1	H2271—C227—C228	118.6
P107—C110—H1103	110.1	C223—C228—C227	119.5 (7)
H1101—C110—H1103	108.3	C223—C228—H2281	119.7
H1102—C110—H1103	108.1	C227—C228—H2281	120.7
Sn1—C111—C112	120.1 (5)	H101—N10—H102	108.9
Sn1—C111—C116	121.8 (4)	H101—N10—C11	108.9
C112—C111—C116	118.0 (5)	H102—N10—C11	109.5
C111—C112—H1121	120.9	H101—N10—C15	107.9
C111—C112—C113	120.7 (6)	H102—N10—C15	109.9
H1121—C112—C113	118.4	C11—N10—C15	111.8 (6)
C112—C113—H1131	119.1	N10—C11—H111	107.9
C112—C113—C114	120.8 (6)	N10—C11—H112	108.6
H1131—C113—C114	120.1	H111—C11—H112	108.9
C113—C114—H1141	120.8	N10—C11—C12	113.2 (6)
C113—C114—C115	119.2 (7)	H111—C11—C12	106.7
H1141—C114—C115	120.0	H112—C11—C12	111.3
C114—C115—H1151	119.5	C11—C12—H121	106.4
C114—C115—C116	120.1 (7)	C11—C12—H122	105.5
H1151—C115—C116	120.4	H121—C12—H122	109.5
C111—C116—C115	121.2 (6)	C11—C12—C13	119.9 (7)
C111—C116—H1161	118.5	H121—C12—C13	109.5
C115—C116—H1161	120.3	H122—C12—C13	105.7
Sn1—C117—C118	122.1 (4)	C12—C13—H131	107.9
Sn1—C117—C122	121.1 (4)	C12—C13—H132	111.4
C118—C117—C122	116.7 (5)	H131—C13—H132	109.5
C117—C118—H1181	119.7	C12—C13—C14	109.50 (2)
C117—C118—C119	122.0 (6)	H131—C13—C14	108.1
H1181—C118—C119	118.3	H132—C13—C14	110.4
C118—C119—H1191	119.8	C13—C14—H141	110.3
C118—C119—C120	120.7 (6)	C13—C14—H142	109.0
H1191—C119—C120	119.5	H141—C14—H142	109.5
C119—C120—H1201	120.6	C13—C14—H143	109.1
C119—C120—C121	119.9 (6)	H141—C14—H143	109.5
H1201—C120—C121	119.5	H142—C14—H143	109.5
C120—C121—H1211	119.5	N10—C15—H151	107.3
C120—C121—C122	119.9 (7)	N10—C15—H152	109.6
H1211—C121—C122	120.6	H151—C15—H152	109.9
C121—C122—C117	120.7 (6)	N10—C15—C16	110.9 (6)
C121—C122—H1221	120.0	H151—C15—C16	108.3
C117—C122—H1221	119.3	H152—C15—C16	110.9
Sn1—C123—C124	120.8 (4)	C15—C16—H161	107.8
Sn1—C123—C128	121.1 (4)	C15—C16—H162	107.4
C124—C123—C128	118.0 (5)	H161—C16—H162	108.7
C123—C124—H1241	119.0	C15—C16—C17	104.9 (7)
C123—C124—C125	121.7 (6)	H161—C16—C17	112.7
H1241—C124—C125	119.3	H162—C16—C17	114.9
C124—C125—H1251	120.8	C16—C17—H171	109.2
C124—C125—C126	119.6 (7)	C16—C17—H172	109.7
H1251—C125—C126	119.6	H171—C17—H172	109.5
C125—C126—H1261	119.8	C16—C17—C18	109.50 (2)
C125—C126—C127	119.7 (6)	H171—C17—C18	109.3
H1261—C126—C127	120.5	H172—C17—C18	109.7
C126—C127—H1271	120.4	C17—C18—H181	108.4
C126—C127—C128	120.4 (7)	C17—C18—H182	110.2
H1271—C127—C128	119.2	H181—C18—H182	109.5
C127—C128—C123	120.5 (6)	C17—C18—H183	109.8
C127—C128—H1281	120.2	H181—C18—H183	109.5
C123—C128—H1281	119.3	H182—C18—H183	109.5
O201—Sn2—O206	174.15 (14)	H201—N20—H202	109.4
O201—Sn2—C211	90.47 (17)	H201—N20—C21	109.2
O206—Sn2—C211	89.08 (18)	H202—N20—C21	106.7
O201—Sn2—C217	85.41 (16)	H201—N20—C25	109.0
O206—Sn2—C217	89.91 (18)	H202—N20—C25	106.6
C211—Sn2—C217	122.4 (2)	C21—N20—C25	115.8 (5)
O201—Sn2—C223	96.22 (19)	N20—C21—H211	108.4
O206—Sn2—C223	89.10 (19)	N20—C21—H212	109.2
C211—Sn2—C223	119.0 (2)	H211—C21—H212	108.5
C217—Sn2—C223	118.6 (2)	N20—C21—C22	111.4 (5)
Sn2—O201—P202	140.8 (2)	H211—C21—C22	110.8
O201—P202—O203	107.4 (2)	H212—C21—C22	108.4
O201—P202—O204	114.2 (2)	C21—C22—H221	106.6
O203—P202—O204	111.8 (2)	C21—C22—H222	109.4
O201—P202—C205	108.9 (3)	H221—C22—H222	109.1
O203—P202—C205	105.6 (3)	C21—C22—C23	115.9 (5)
O204—P202—C205	108.4 (3)	H221—C22—C23	107.7
P202—O203—H2031	122.9	H222—C22—C23	107.9
P202—C205—H2051	110.5	C22—C23—H231	109.5
P202—C205—H2052	110.7	C22—C23—H232	109.1
H2051—C205—H2052	109.5	H231—C23—H232	109.6
P202—C205—H2053	109.7	C22—C23—C24	109.50 (2)
H2051—C205—H2053	108.1	H231—C23—C24	109.4
H2052—C205—H2053	108.3	H232—C23—C24	109.6
Sn2—O206—P207	140.5 (2)	C23—C24—H241	108.3
O206—P207—O208	110.3 (3)	C23—C24—H242	111.1
O206—P207—O209	113.7 (2)	H241—C24—H242	109.2
O208—P207—O209	109.1 (2)	C23—C24—H243	110.6
O206—P207—C210	109.6 (3)	H241—C24—H243	109.2
O208—P207—C210	102.7 (3)	H242—C24—H243	108.4
O209—P207—C210	110.9 (3)	N20—C25—H251	107.4
P207—O208—H2081	119.1	N20—C25—H252	108.2
P207—C210—H2101	110.7	H251—C25—H252	108.6
P207—C210—H2102	110.7	N20—C25—C26	111.0 (5)
H2101—C210—H2102	108.5	H251—C25—C26	110.3
P207—C210—H2103	110.3	H252—C25—C26	111.2
H2101—C210—H2103	108.3	C25—C26—H261	109.2
H2102—C210—H2103	108.3	C25—C26—H262	108.7
Sn2—C211—C212	120.5 (4)	H261—C26—H262	108.7
Sn2—C211—C216	121.6 (5)	C25—C26—C27	112.8 (5)
C212—C211—C216	117.9 (6)	H261—C26—C27	108.0
C211—C212—H2121	121.0	H262—C26—C27	109.4
C211—C212—C213	120.2 (6)	C26—C27—H271	108.6
H2121—C212—C213	118.8	C26—C27—H272	108.2
C212—C213—H2131	119.1	H271—C27—H272	110.1
C212—C213—C214	121.7 (6)	C26—C27—C28	114.4 (6)
H2131—C213—C214	119.2	H271—C27—C28	107.1
C213—C214—H2141	120.6	H272—C27—C28	108.4
C213—C214—C215	119.5 (6)	C27—C28—H281	109.6
H2141—C214—C215	119.9	C27—C28—H282	112.2
C214—C215—H2151	119.8	H281—C28—H282	108.6
C214—C215—C216	118.8 (7)	C27—C28—H283	108.0
H2151—C215—C216	121.4	H281—C28—H283	109.1
C215—C216—C211	121.8 (7)	H282—C28—H283	109.5

### Hydrogen-bond geometry (Å, º)

**Table:**

*D*—H···*A*	*D*—H	H···*A*	*D*···*A*	*D*—H···*A*
O103—H1031···O109^i^	0.85	1.73	2.582 (10)	180 (1)
O108—H1081···O104^ii^	0.85	1.64	2.490 (10)	180 (1)
C122—H1221···O101	0.92	2.36	2.976 (10)	124 (1)
O203—H2031···O209^iii^	0.85	1.63	2.478 (10)	180 (1)
O208—H2081···O204^iv^	0.85	1.71	2.561 (10)	180 (1)
C228—H2281···O206	0.92	2.35	2.961 (10)	124 (1)
N10—H101···O209^iii^	0.89	1.90	2.772 (10)	165 (1)
N10—H102···O109	0.90	1.92	2.777 (10)	160 (1)
C15—H152···O203	0.98	2.45	3.134 (10)	127 (1)
C16—H161···O103^ii^	0.97	2.51	3.418 (10)	156 (1)
N20—H201···O204^iv^	0.89	1.95	2.780 (10)	154 (1)
N20—H202···O104	0.90	1.88	2.756 (10)	167 (1)
C25—H252···O108^i^	0.97	2.51	3.212 (10)	129 (1)
C26—H262···O208	0.98	2.52	3.302 (10)	138 (1)

Symmetry codes: (i) −*x*+1, *y*+1/2, −*z*+1/2; (ii) −*x*+1, *y*−1/2, −*z*+1/2; (iii) −*x*+2, *y*−1/2, −*z*+1/2; (iv) −*x*+2, *y*+1/2, −*z*+1/2.
